# TiO_2_ Nanotubes Functionalized with Icariin for an Attenuated In Vitro Immune Response and Improved In Vivo Osseointegration

**DOI:** 10.3390/jfb13020043

**Published:** 2022-04-14

**Authors:** Andreea-Mariana Negrescu, Valentina Mitran, Wanda Draghicescu, Simona Popescu, Cristian Pirvu, Iuliana Ionascu, Teodoru Soare, Seralp Uzun, Sorin Mihai Croitoru, Anisoara Cimpean

**Affiliations:** 1 Department of Biochemistry and Molecular Biology, Faculty of Biology, University of Bucharest, 91-95 Splaiul Independentei, 050095 Bucharest, Romania; andreea.mariana.negrescu@drd.unibuc.ro (A.-M.N.); valentina.mitran@bio.unibuc.ro (V.M.); 2 Faculty of Chemical Engineering and Biotechnology, University Politehnica of Bucharest, 1-7 Polizu, 011061 Bucharest, Romania; wanda.polipciuc@chimie.upb.ro (W.D.); simona.popescu@upb.ro (S.P.); cristian.pirvu@upb.ro (C.P.); 3 Faculty of Medical Engineering, University Politehnica of Bucharest, 1-7 Polizu, 011061 Bucharest, Romania; 4 Faculty of Veterinary Medicine, University of Agricultural Sciences and Veterinary Medicine, 105 Spl. Independentei, 050097 Bucharest, Romania; driulianaionascu10@gmail.com (I.I.); teodoru.soare@gmail.com (T.S.); seralp.uzun@gmail.com (S.U.); 5 Machines and Manufacturing Systems Department, University Politehnica of Bucharest, 313 Spl. Independentei, 060042 Bucharest, Romania; sorin.croitoru@gmail.com

**Keywords:** TiO_2_ nanotubes, icariin, drug delivery platform, immune response, bone fracture healing

## Abstract

Due to their superior mechanical and chemical properties, titanium (Ti) and its alloys have been widely used as orthopedic implantable devices. However, their bioinertness represents a limitation, which can be overcome by employing various surface modifications, such as TiO_2_ nanotube (TNT) fabrication via electrochemical anodization. Anodic TNTs present tunable dimensions and unique structures, turning them into feasible drug delivery platforms. In the present work, TNTs were loaded with icariin (Ica) through an adhesive intermediate layer of polydopamine (DP), and their in vitro and in vivo biological performance was evaluated. The successful fabrication of the modified surfaces was verified by scanning electron microscopy (SEM), atomic force microscopy (AFM), Fourier transform infrared spectroscopy (FTIR), and contact angle measurements (CA), while the in vitro release of Ica was evaluated via UV-VIS spectrophotometry. In terms of in vitro behaviour, comparative studies on RAW 264.7 macrophages demonstrated that the TNT substrates, especially TNT-DP-Ica, elicited a lower inflammatory response compared to the Ti support. Moreover, the in vivo implantation studies evinced generation of a reduced fibrotic capsule around this implant and increased thickness of the newly formed bone tissue at 1 month and 3 months post-implantation, respectively. Overall, our results indicate that the controlled release of Ica from TNT surfaces could result in an improved osseointegration process.

## 1. Introduction

As a direct consequence of human lifespan extension and an increase in activity levels, especially at later stages in life, the need for biomaterials capable of restoring damaged bone structure and function continues to increase dramatically. For many years, titanium (Ti) and titanium-based alloys have represented the very first choice in metallic biomaterials for various biomedical applications, e.g., for implants and prosthesis production in orthopaedics, and dental as well as maxillofacial surgery, as they possess the perfect combination of characteristics, such as favourable biocompatibility, high corrosion resistance, satisfactory tensile strength, and an adequate flexibility [1,2]. The high biocompatibility of Ti is attributed to its bioinert state and low electrical conductivity, which in turn results in surface electrochemical oxidation leading to the formation of TiO_2_—a thin passive oxide layer that enhances the corrosion resistance of the biomaterial [3]. However, despite the numerous advantages, Ti-based biomaterials exhibit a reduced hardness, which in turn results in a low abrasion and wear resistance, thus limiting the service life of the implant to almost 15 years [4]. Likewise, research studies conducted in the last years have made transparent the fact that the fate of the implant depends altogether on the biocompatibility and surface characteristics of the aforementioned biomaterials [4]. Therefore, in order to achieve rapid osseointegration and an enhancement of the in vivo performance of dental and orthopedic Ti implants, an extensive variety of approaches to modifying their surface has been carried out [5]. Amongst those strategies, surface functionalization with various bioactive materials and molecules has been reported as a practical method of overcoming the poor osteoinductive characteristics of Ti implants [6]. One of the most extensively investigated biological modifications is represented by the incorporation of various bioactive agents onto the surface of the implant, with the aim of improving bone tissue reconstruction ability [7]. Moreover, rapid developments in the nano-/microtechnology field have led to an increase in the design and use of implantable devices with nano-/micro- surface characteristics for a more favourable osseointegration process [7]. For example, a large body of studies has demonstrated that titania nanotubes (TNTs) obtained through the anodization process represent a viable means to improve bone regeneration [8] and prevent inflammation [9,10]. Moreover, TiO_2_ nanostructures possess great potential as favourable candidates for a wide range of medical applications, with numerous research studies focused solely on designing and developing nanostructures capable of diagnosing, monitoring, repairing and improving human health [11,12,13]. In addition, TNTs have been proven to be attractive platforms for the delivery of a broad spectrum of bioactive molecules involved in bone tissue regeneration [14,15,16], mostly proteins such as bone morphogenic proteins (BMP)-2 [17,18], epidermal growth factor (EGF) [19], fibroblast growth factor (FGF) [20], and platelet-derived growth factor (PDGF) [21]. However, the high associated costs, short half-life and potential side effects restrict the clinical applications of these bioactive agents. Hence, plant-derived natural compounds can offer a promising alternative due to their lower cost and potentially higher safety. In this context, the present study offers new insight into the fabrication of TNT surfaces functionalized with icariin (Ica), through an adhesive intermediate layer of polydopamine (DP) as a means to improve Ti implant osseointegration. The choice of Ica, the main active ingredient of *Herba Epimedii*, is motivated by previous reports showing its positive effects on pre-osteoblast proliferation, differentiation, extracellular matrix mineralization and the expression of bone-related genes and proteins [22,23,24,25,26]. Moreover, Ica has been demonstrated to decrease the movement of osteoclasts and their resorptive activity [27,28], and, furthermore, to increase the angiogenic process through the stimulation of endothelial cell migration, proliferation, and tubulogenesis [29]. Furthermore, Ica proved its potential as a post-infection treatment for bone infection [30]. In addition, the combination of nanotubular surfaces with biodegradable polymer- based coatings has emerged in the last years as a promising approach for achieving a better performance for controlled drug release [31,32]. The use of polydopamine as an intermediate bio-adhesive layer has several advantages, such as a low cost, facile deposition onto various types of materials, strong reactivity for secondary functionalization with bioactive molecules, good biocompatibility, and stability [33,34,35]; thus, DP can be used as an efficient modification strategy for TNT surfaces, laying the base structure for the subsequent immobilization of Ica.

Up to now, numerous endeavours have been made in order to improve the clinical application of ceramic materials, such as calcium phosphate cement [36,37], hydroxyapatite [38,39] and tricalcium phosphate [40,41], by adding Ica. Very recently Ica has been also used to modify PLLA/chitosan composite fibrous membranes exhibiting favourable osteogenic activity and superior angiogenic activity [42]. However, only a few studies have used the incorporation of Ica into Ti implants [43,44,45]. Overall, the results reported in the literature underline the favourable outcome of Ica as a surface functionalization agent in terms of bone regeneration. However, despite the increasing use of Ica, data reporting the immunomodulatory effects of Ica-modified biomaterials are scarce. In addition, most in vivo studies have been undertaken for short periods of time. Accordingly, the present study aims to evaluate and compare the in vitro inflammatory response of macrophages and in vivo bone regeneration following the application of bare Ti, and TiO_2_ nanotube-modified Ti implants, without and with Ica functionalization, for up to 12 weeks in a rat femoral defect model.

## 2. Materials and Methods

### 2.1. Materials

#### 2.1.1. TiO_2_ Nanotube Fabrication

Prior to the TiO_2_ nanotube fabrication, commercial Ti plates (1 × 1 cm, 0.89 mm thick, 99.7% purity) provided by Alfa Aesar Co. (Tianjin, China) were mechanically polished with silicon-carbide sandpaper of increasing grit sizes (800 up to 1200) and cleaned in purified water, ethanol and acetone for 15 min. The TiO_2_ nanotubes were fabricated via the anodic oxidation method, in an electrolyte containing ethylene glycol (Chimreactiv SRL Co., Bucharest, Romania), NH_4_F (Sigma-Aldrich Co., Steinheim, Germany)—0.5 wt.%, and ultrapure water (2% *v*/*v*). This process took place in a two-electrode electrochemical cell that used a platinum plate as a counter electrode and a Ti plate as the working electrode, with both being connected to a current source MPS-6005L-2-MATRIX Co. The anodization process was realised for 2 h at an increasing voltage, from 0 to 30 V, with 2 V/10 s, as previously described [46]. The resulting anodized samples were thoroughly rinsed with purified water and annealed (CALORIS Microterm 1206, Bucharest, Romania). The annealing method was performed at a temperature of 450 °C, for 3 h, with a heating rate of 5 °C/min, and the cooling was done overnight at room temperature.

#### 2.1.2. Functionalization of Ti/TiO_2_ Nanotubes with Icariin

In the first phase, the DP adhesive intermediate layer was deposited on the TNT substrates (TNT-DP) at room temperature via immersion in an alkaline solution that contained DP in a concentration of 1 mg/mL in TRIS (0.1 mol/L), at pH 8.5, for 24 h, under magnetic stirring, and without light exposure. Finally, the DP-coated nanotubular samples were washed with sterile-filtered Milli-Q water and dried on filter paper in the absence of light [46]. In the second phase, the immobilization of Ica on the newly fabricated nanostructures took place via physical adsorption. A 500 µM Ica solution in ethanol was prepared from a stock solution of 50 mg/mL of Ica (Sigma-Aldrich, Steinheim, Germany) in dimethyl sulfoxide (DMSO) (Sigma Aldrich, Steinheim, Germany). Then, a total of 200 µL of sterile Ica solution was pipetted onto the TNT-DP surface and allowed to dry in a sterile hood. After the last loading cycle, the samples were quickly rinsed in a phosphate-buffered saline (PBS, Life Technologies Corporation, Grand Island, NY, USA) solution and afterwards used as substrates in cell culture studies in parallel with flat Ti and bare TNT samples.

#### 2.1.3. TiO_2_ Nanotube Characterization

To characterize the morphology of the coated samples, a field-emission scanning electron microscope (SEM, FEI/Philips XL-30 QUANTA 650, FEI Company, Eindhoven, The Netherlands) was used. The surface roughness was determined with an Atomic Force Microscope (AFM, APE Research, Trieste, Italy) in contact mode and the obtained data were processed with Gwyddion 2.9 software. To evaluate the hydrophilic/hydrophobic balance, the contact angles of the resulting samples were measured using a 100 Optical Contact Angle Meter—CAM 100, with an accuracy of ±1°, at room temperature and light. Furthermore, the contact angle values were obtained using three liquids with various polarities: distilled water, ethylene glycol, and di-methyl sulfoxide (Sigma-Aldrich, Steinheim, Germany) in order to quantify the surface free energy. The method for calculating the surface energy was detailed in a previous paper [47]. In addition, the electrochemical stability evaluation was conducted in a NaCl 0.9% solution using an Autolab PGSTAT 302N potentiostat/galvanostat (Metrohm Autolab B.V., Utrecht, The Netherlands).

#### 2.1.4. In Vitro Release of Icariin

The in vitro release of Ica from the loaded substrates was evidenced at the predetermined time points of 1, 2, 4, 6 h; and 1, 2, 4, 7, and 14 days in a PBS solution (pH 7.4). Afterwards, the adsorption was read at 270 nm by means of a UV-Vis spectrophotometer (Jasco V-530 UV/Vis, Jasco Corporation, Tokyo, Japan). The percentage of drug released was calculated by dividing the accumulated amount of the released drug by the total drug-loading amount. The total drug loading quantity was the amount of drug released when the UV–Vis absorbance did not change anymore, at the end of the experiment. This investigation was performed in triplicate for each time point.

### 2.2. Cell Culture

The in vitro experiments were conducted on macrophage cell line RAW 264.7 (American Type Culture Collection, Manassa, VA, USA). These cells were seeded onto the prepared specimens at a density of 10^5^ cells/cm^2^ and maintained for 24 h in a humidified atmosphere of 5% CO_2_ at 37 °C in Dulbecco’s Minimal Essential Medium (DMEM, Sigma-Aldrich Co., St. Louis, MO, USA) supplemented with 10% (*v*/*v*) heat-inactivated fetal bovine serum (FBS, Life Technologies Corporation, Grand Island, NY, USA) and 1% (*v*/*v*) penicillin (10,000 units/mL)/streptomycin (10 mg/mL) (Sigma-Aldrich Co., St. Louis, MO, USA). The experiments were simultaneously performed in standard culture conditions and under pro-inflammatory stimulation (medium supplementation with 100 ng/mL *Escherichia coli* lipopolysaccharide (LPS, Sigma-Aldrich Co., St. Louis, MO, USA)).

### 2.3. Live/Dead Assay

In order to assess the survival rate of the RAW 264.7 macrophages seeded on the tested supports, the Live/Dead Cell Viability/Cytotoxicity Assay Kit (Molecular Probes, Eugene, OR, USA) was used after 1 day and 3 days in culture as previously described [9]. Summarily, the growth medium was discarded, and the cells were washed with PBS (Life Technologies Corporation, Grand Island, NY, USA), followed by an incubation with a mixture of 2 µM calcein AM (acetoxymethyl) and 4 µM ethidium homodimer-1 (EthD-1) for 10 min in the dark. In the end, the stained cells were visualised, and representative images were taken with an acquisition system (Cell F, Version 5.0) under an inverted fluorescence microscope (Olympus IX71, Olympus, Tokyo, Japan).

### 2.4. CCK-8 Assay

The macrophages’ metabolic activity was investigated at 24 h post-seeding by means of Cell Counting Kit 8 (CCK-8, Sigma-Aldrich Co., St. Louis, MO, USA) in accordance with the manufacturer’s instructions. Thus, the growth medium was removed and replaced with a mixture of fresh culture medium containing 10% CCK-8 reagent, and then the cells were incubated for an additional 2 h at 37 °C in a humidified 5% CO_2_ atmosphere. Finally, the absorbance at 450 nm (OD 450) was measured using a microplate reader (FlexStation 3, microplate reader, Molecular Devices, San Jose, CA, USA).

### 2.5. Cell Morphology Analysis

To study the attachment, spreading and morphology of the RAW 264.7 macrophages seeded on the tested surfaces, the fluorescent staining of actin cytoskeleton with Alexa Flour 546-conjugated phalloidin was performed. Briefly, at the end of the experimental periods of 24 h and 72 h, the adherent cells were fixed with 4% paraformaldehyde and permeabilized and blocked with a solution of 0.1% Triton X-100/2% bovine serum albumin for 15 min, at room temperature. Afterwards, the macrophage-populated samples were incubated with Alexa Fluor 546 Phalloidin (20 µg mL^−1^, Invitrogen, Eugene, OR, USA) for 15 min at room temperature. Then, after three washes with PBS, the samples were visualised with an Olympus IX71 inverted microscope (Olympus IX71, Olympus, Tokyo, Japan) and relevant fields were captured using the Cell F software (Version 5.0).

### 2.6. Quantification of Secreted Cytokines and Chemokines

To determine the protein levels of cytokines such as tumour necrosis factor (TNF)-α, interleukin (IL)-6 and chemokines (monocyte chemotactic protein (MCP)-1) secreted into the culture medium at 24 h post-seeding, the cell culture supernatants were subjected to the enzyme-linked immunosorbent assay (ELISA) by using specific kits (R&D Systems, Minneapolis, MN, USA) according to package insert instructions. The absorbance of the final products was measured with a microplate reader (FlexStation 3 microplate reader, Molecular Devices, San Jose, CA, USA) and the corresponding concentrations were expressed in pg mL^−1^ by reporting their standard curve.

### 2.7. Nitric Oxide Assay

The amount of nitric oxide (NO) released by the RAW 264.7 macrophages and accumulated into the culture medium after 24 h of culture was detected following reaction with Griess reagent (Promega, Madison, WI, USA), as previously reported [9]. The absorbance at 550 nm of the resulted dye enabled the determination of the final concentration of nitrite in the samples.

### 2.8. In Vivo Implantation and Histological Analysis

#### 2.8.1. Implant Preparation and Surgery

For the in vivo animal studies, Ti plates with a length of 1 × 1 cm were used to fabricate implantable pins (diameter: 2 mm; length: 16 mm) through a sequential turning process on a fine mechanics lath with a normal lath cutter made of High-Speed Steel (HSS), as previously described [48]. In summary, after reaching the desired length, the turning bars were cut off, and the resulting implants were ultrasonically washed with an anti-grease detergent solution (1:10 parts volume). Then, the implants were rinsed in clean water, treated with water steam pressure, and finally dried with air pressure. In the end, their surface was modified via anodic oxidation and the resulting TiO_2_ nanotubes were functionalised with Ica through an intermediate DP layer, as described in Section 2.1.

To perform in vivo implantation, 4-month-old male Wistar rats (mean body weight of 0.250 kg) were used. It is worth mentioning that all of the experimental procedures were approved by the Bioethics Committee of the University of Agronomical Sciences and Veterinary Medicine of Bucharest (Approval code No. 11/08.02.2018). For the surgical approach, 3 rats per time period were used, so that each rat could receive in its two posterior legs a different type of implant. The rats were anesthetized by the intraperitoneal administration of a combination of ketamine and dexdomitor in sterile saline solution [48]. The narcotic state was maintained by mask inhalation of isoflurane vaporized at concentrations of 1–1.5 vol.%. After shaving the hair of and disinfecting the surgery area, a 3 cm longitudinal incision was made on the external side of the thigh to cut open the skin, and the dissection of the muscle was performed. Afterwards, at the lateral surface of the femur in the middle third of the diaphysis, a 0.5 cm long, linear, longitudinal fracture was performed in the compact bone, using a Dental Unit cutter. Through the newly created fracture, the implant was inserted into the medullary canal of both posterior legs. The implantation site was covered with the adjoining muscle tissue and sutured using absorbable (PDS 3/0) threads in separate points, while the skin was sutured with non-absorbable (nylon 4/0) threads in simple continuous pattern. Then, the rats received antibiotics (Enroxil—10 mg/kg BW) and anti-inflammatory drugs (Metacam—0.2 mg/kg BW) by subcutaneous injection for a period of 6 days. X-ray analysis of the surgical areas was performed directly after surgery in order to highlight the presence of the implants. Moreover, during the experimental period of up to 90 days, the animals were maintained under close observation without mortality or morbidity being noticed. At the end of each experimental time point, in order for the femur to be removed by disarticulation, the rats were subjected to the same premedication, and the euthanasia was performed by the intraperitoneal administration of 0.5 mL of T61.

#### 2.8.2. Histological Preparation and Procedures

At the end of the experimental periods, the rats were euthanized, and the harvested bone specimens were processed as previously shown [48]. For this, they were subjected to a fixation procedure in 10% buffered formaldehyde solution for 48 h, followed by a 20 day-immersion in a decalcified solution (ethylenediaminetetraacetic acid) and paraffin wax embedment. Sections of 3–4 µm in thickness were prepared by using a saw microtome and then classically stained with haematoxylin and eosin (HE) for the histological examination with an Olympus BX41 microscope. Representative images were captured using an Olympus DP25 Camera (Cell B Software, Version 2.3).

### 2.9. Statistical Analysis

Data were analysed using the Graph Prism software (Version 6, GraphPad, San Diego, CA, USA) using one-way ANOVA with Bonferroni’s multiple comparison tests. Data are expressed as the mean ± standard deviation (SD). For all of the tests, statistical significance was considered for *p* values lower than 0.05.

## 3. Results

### 3.1. Surface Characterization

#### 3.1.1. Ti/TiO_2_ Characterization

**Figure 1:**
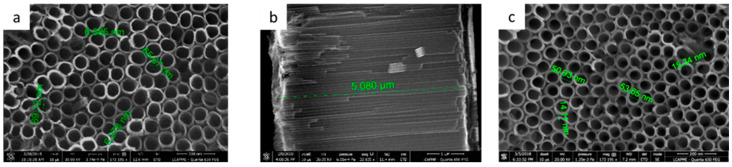
Representative scanning electron micrographs of TiO_2_ nanotubes (TNT) obtained on Ti substrate, after anodic oxidation, in an electrolyte containing ethylene glycol, NH4F—0.5 wt.%, ultrapure water (2% *v*/*v*) at 30 V, before and after polydopamine self-deposition: (**a**) top surface for simple TNT—scale bar 200 nm; (**b**) cross section for TNT coating—scale bar 2 µm, and (**c**) TNT-DP sample—top surface for nanotubes after their immersion in the dopamine solution—scale bar 200 nm. The inner diameter of nanotubes decreased from 65–70 nm to 50–55 nm after DP deposition on the tube walls.

Figure 1 illustrates the surface morphology of the uncoated and DP-coated TiO_2_ nanotubes observed by SEM. By optimizing the anodization conditions, the obtained TiO_2_ nanotubes were uniform and well-organized, with an inner diameter of around 65–70 nm, a wall thickness of 6–7 nm (Figure 1a), and a TiO_2_ layer thickness of about 5 µm (Figure 1b). In the case of the coated surfaces, SEM images indicated a well-kept surface nano-architecture, suggesting that the deposition of a uniform layer of DP did not obstruct the newly formed nanotubes. However, the tube diameter was reduced to 50–55 nm, as a direct consequence of DP deposition on the interior of the tube walls. In addition, their thickness increased to 14–15 nm (Figure 1c).

#### 3.1.2. Surface Roughness

**Figure 2:**
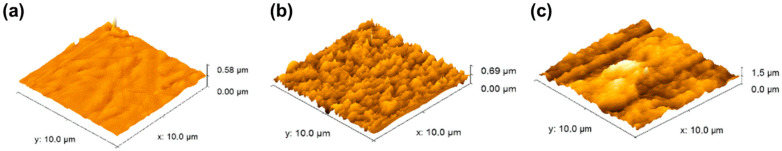
Atomic Force Microscopy (AFM) analysis of surface topography for (**a**) unmodified Ti; (**b**) TNT; (**c**) TNT-DP-Ica. The roughness increased in the following order: Ti (54 nm) < TNT (95 nm) < TNT-DP-Ica (159 nm).

The surface topography of the tested supports was observed by atomic force microscopy (AFM), and Figure 2 shows examples of AFM images obtained from the uncoated (Figure 2a) and coated (Figure 2b,c) TNT surfaces. From the AFM images, it could be observed that nanostructuring led to an increase in surface roughness from 54 nm for Ti to 95 nm for TiO_2_ nanotubes. Moreover, after DP surface functionalization and Ica loading, the final surface roughness reached a value of 159 nm, suggesting that surface functionalization could influence surface topography by increasing its roughness, and indirectly affecting cell behavior with regards to cell spreading and morphology.

#### 3.1.3. Surface Energy Determination

Similar to the topography of the surface, its wettability represents another important parameter capable of influencing the cell’s biological response in terms of protein adsorption and adhesion. Protein adhesion to certain surfaces can involve large energy scales, dynamic conformational alterations, and reorientation as a response to surface topography [49]. Table 1 shows the surface energies for the untreated and modified surfaces determined by the average values of the contact angles. By analysing the obtained values for the contact angle, it can be observed that after surface nanostructuring, the degree of the hydrophilic character was increased. Moreover, the presence of the DP film led to an insignificant increase in contact angle values, probably as a direct consequence of –OH groups reduction after its grafting to the TNT surface [46]. Regarding the surface energy, as can be observed, the values changed from ~40 mJ/m^2^ in the case of Ti to ~70 mJ/m^2^ for the TNT supports. However, when the surface was functionalized with Ica, the surface energy was not significantly altered (~60 mJ/m^2^), especially as this compound will be progressively released and diffuse in time into the adjacent tissue. Therefore, it is not a determining factor when assessing the degree of wettability. Thus, it can be argued that the final value of surface energy is determined mainly by the way the surface nanostructure performed and less by the presence of the DP-Ica coating.

#### 3.1.4. Electrochemical Impedance Spectroscopy

**Figure 3:**
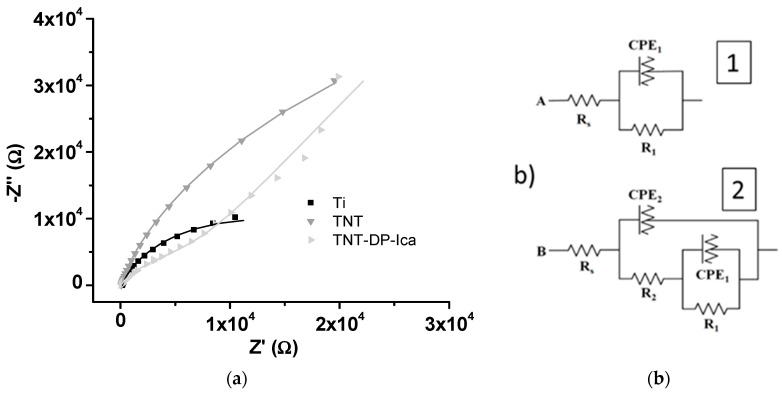
Electrochemical Impedance Spectra for determination of electrochemical behaviour of Ti, TNT, TNT-DP-Ica samples analysed in NaCl 0.9% solution: (**a**) Nyquist diagram; symbols—experimental data, lines—fitting results; (**b**) equivalent circuits used for fitting EIS data (1) for Ti and (2) for TNT and TNT-DP-Ica samples.

To establish the electrochemical behaviour of the tested samples, electrochemical impedance spectroscopy (EIS) was performed. In Figure 3, the Nyquist diagrams and equivalent circuits proposed for data fitting are presented. As can be observed from Figure 3b, two different electrical circuits were proposed: one for the unmodified Ti sample (1) and another one for the TNT and TNT-DP-Ica supports (2).

The values of resistance obtained by data fitting are presented in Table 2.

The resistance, R_1,_ corresponding to the native TiO_2_ layer, is in the order of hundreds kΩ for all three samples representing the layer with the most important contribution to corrosion resistance. The deposition of TiO_2_ in the form of nanotubes introduced a supplementary lower resistance, R_2_, about 3 kΩ, due to the open nanotubular oxide structure. Moreover, the deposition of DP increased the values of R_2_ up to 12 kΩ, probably due to the non-conducting properties of the DP deposited onto the TiO_2_ nanostructured layer. Thus, by surface nanostructuring and by functionalizing the surface of the materials, the corrosion resistance of the samples was greatly improved. The results are similar to those obtained in a previous paper [35].

#### 3.1.5. In Vitro Release of Icariin

**Figure 4:**
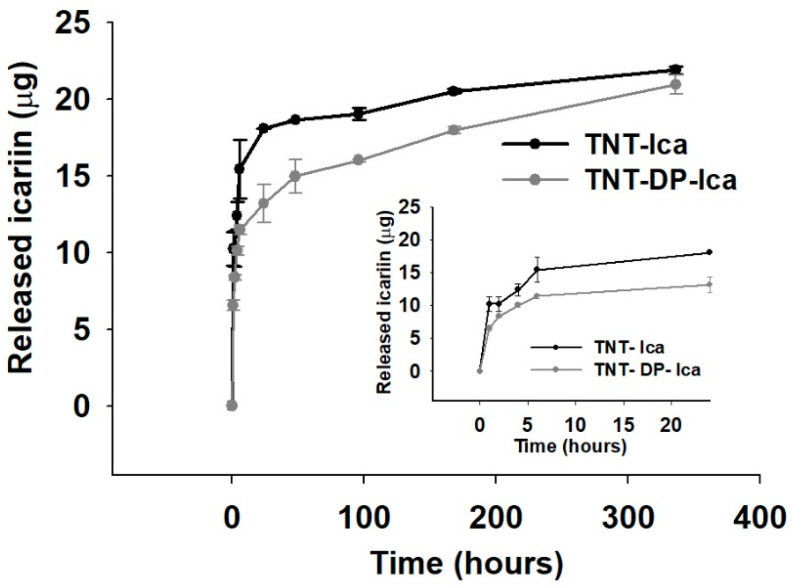
The in vitro release of Ica-loaded samples (TNT-Ica and TNT-DP-Ica), monitored at the predetermined time points of 1, 2, 4, 6 h; and 1, 2, 4, 7, and 14 days in a PBS solution (pH 7.4). Results are presented as means ± SD. For each interval of time, three samples were tested. After 7 days, the Ica released exhibited by TNT-DP-Ica was still in progress, due to the better linking properties of polydopamine.

Figure 4 shows that, within the first hour, Ica was rapidly released, with a rate of 10 µg/h for the TNT substrate, and 6.5 µg/h for the TNT-DP sample. However, for the next 5 h (Figure 4, inset), the slope of the release profile was diminished to a rate of 5 µg/h for both analysed samples. These first release steps were then followed by a slow Ica release: 0.03 µg/h for TNT and 0.04 µg/h for TNT-DP, respectively. In the last 168 h, the drug releasing profile for the TNT support was almost completely flat, while for the TNT-DP sample, the Ica release was still in progress, thus revealing that the DP film presented better linking properties in comparison to the bare TNT surface.

### 3.2. In Vitro Responses of Macrophages

#### 3.2.1. Cell Viability and Proliferation

**Figure 5:**
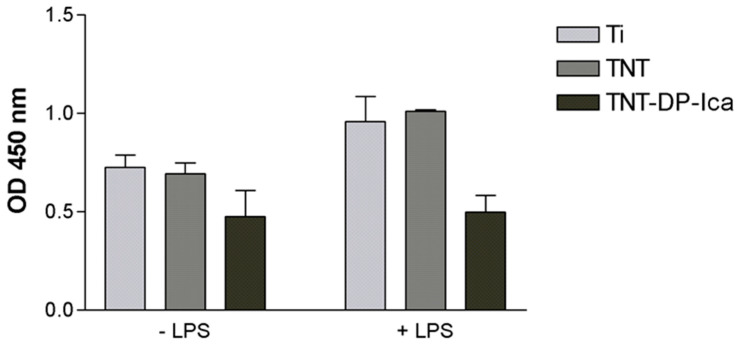
CCK-8 assay highlighting the viability of the RAW 264.7 cells grown in direct contact with the analysed samples (flat Ti; TNT; TNT-DP-Ica) for 24 h, under standard (−LPS) and pro-inflammatory (+LPS) conditions. Data analysis was based on mean ± SD (*n* = 3).

Taking into account the importance of nanoarchitecture in directing the cell behaviour in terms of survival, proliferation and differentiation [50], a first objective of the in vitro studies was to analyse the impact of the tested surfaces on the survival rate of RAW 264.7 macrophages by employing the CCK-8 assay. Figure 5 shows a lower number of viable metabolic active cells grown on the surface of the coated TNT-DP-Ica sample when compared to the flat Ti and bare TNT substrates, in the absence (−LPS) and presence (+LPS) of the pro-inflammatory stimulus, although the statistical analysis did not evince any significant differences. At the same time, no visible differences could be observed between the control surface (flat Ti) and the TNT support in terms of the number of viable cells.

**Figure 6:**
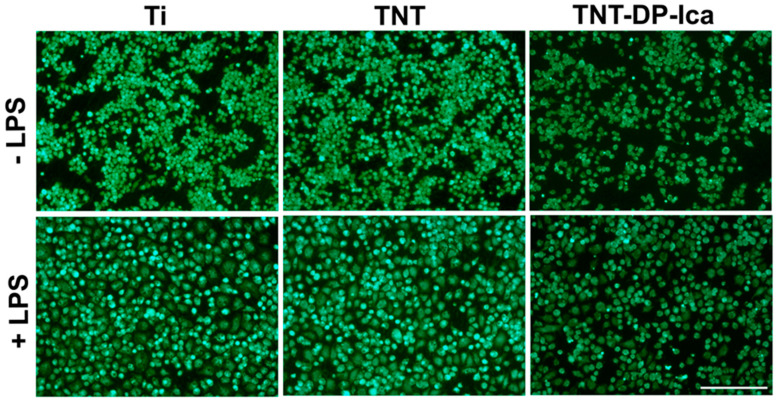
Viability of the RAW 264.7 cells grown directly on the surface of the flat T, TNTs and TNT-DP-Ica supports for 24 h, under standard (−LPS) and inflammatory (+LPS) conditions. The Live & Dead assay highlighted the presence of viable cells (green fluorescence) on all of the tested supports, with no visible red fluorescence specific for dead cells. Scale bar represents 100 µm.

Furthermore, in order to bring forth supporting information for the CCK-8 results, the Live & Dead Cell Viability/Cytotoxicity Assay was performed. The representative fluorescent images (Figure 6) show the ability of the tested surfaces to sustain the survival of the RAW 264.7 macrophages, with no evidence of the presence of red-stained dead cells. However, while no obvious cell death and cell density decrease could be observed on the bare TNT surface, the Ica-loaded support showed a much lower cellular density in both experimental culture conditions, suggesting that even though the Ica-functionalized nanostructured surface did not kill the cells, it significantly inhibited the cell proliferation process.

#### 3.2.2. Cell Morphological Features

**Figure 7:**
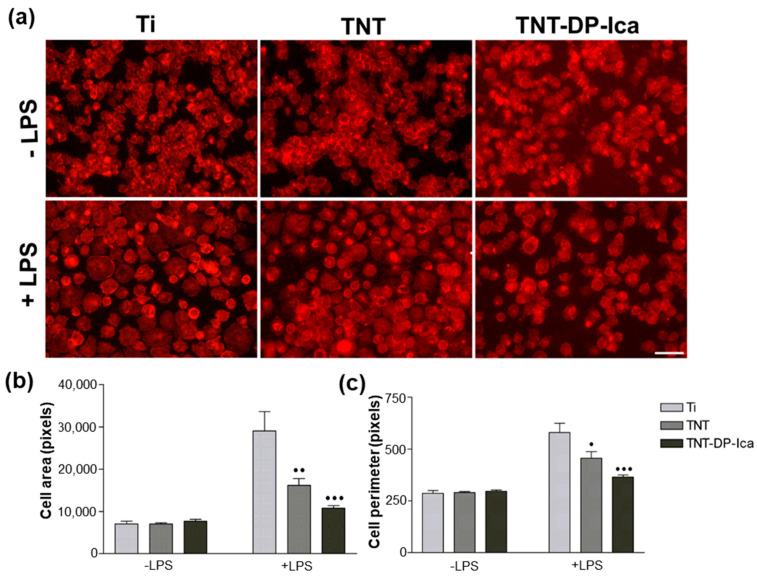
(**a**) Representative fluorescence images of morphological features exhibited by macrophages seeded directly on the surface of the tested samples (flat Ti, TNT, TNT-DP-Ica) after 24 h of culture under standard (−LPS) and pro-inflammatory (+LPS) conditions. Labelling of the actin cytoskeleton with phalloidin conjugated with Alexa Flour 546 showed significant morphological changes between the cells grown on the analysed samples, especially under LPS stimulation, with the nanostructured surfaces being able to attenuate the LPS-induced alternations compared with the flat Ti sample. Scale bar represents 50 µm; (**b**) Quantification of cell spreading area; (**c**) Statistical results of macrophage perimeter. Data analysis was based on mean ± SD (*n* = 20, *• p* < 0.05, *•• p* < 0.01, ••• *p* < 0.001 vs. Ti).

The cellular adhesion and morphology of the RAW 264.7 macrophages on the surface of the tested substrates were investigated by actin cytoskeleton labelling with Alex Flour 546-conjugated phalloidin. As seen in Figure 7a, the cells maintained in standard culture conditions presented a typical rounded morphology on all of the samples. However, upon LPS stimulation, major differences regarding the cells’ morphology were observed among the analysed substrates. Thus, macrophages cultured onto the flat Ti support exhibited an increase in spreading degree, a behaviour which in the specialised literature is associated with an activated and migratory M1 phenotype. On the contrary, the fabricated TiO_2_ nanotubes manifested a protective effect against the morphological alterations caused by the bacterial LPS. Furthermore, the addition of Ica significantly attenuated the morphological changes usually observed in macrophages upon LPS treatment, the great majority of cells displaying a round-shape morphology similar to the one adopted in the absence of the inflammatory agent.

From a quantitative perspective, the RAW 264.7 macrophage-like spread areas and perimeters of the cells were measured and quantified as seen in Figure 7b,c. The spread area and perimeter of the cells grown on tested samples declined in the following order Ti > TNT > TNT-DP-Ica, suggesting that the coated TNT surface could be capable of inhibiting the activation of RAW 264.7 macrophages.

#### 3.2.3. Extracellular Release of Inflammatory Mediators

**Figure 8:**
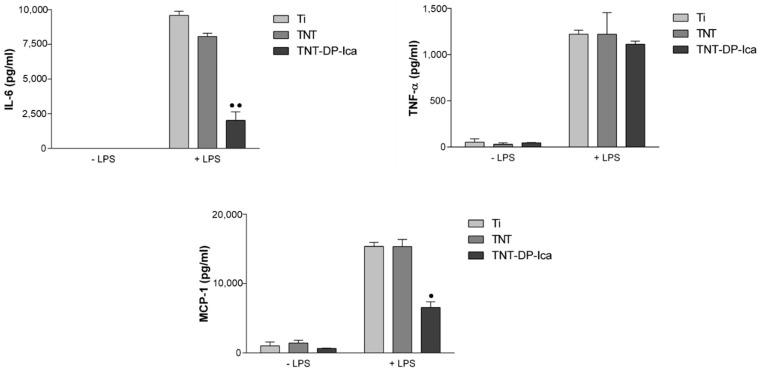
The extracellular levels of IL-6, TNF-α, and MCP-1 accumulated in cell culture medium of macrophages grown for 24 h on the analysed substrates, under standard (−LPS) and pro-inflammatory (+LPS) conditions. In pro-inflammatory (+LPS) conditions, the lowest amounts of cytokines were secreted by the RAW 264.7 cells grown directly on the surface of the Ica-functionalised TNT, while under standard culture conditions (−LPS), very low or non-existing amounts of pro-inflammatory mediators could be quantified. Results are presented as means ± SD (*n* = 3, • *p* < 0.05, *••* *p* < 0.01 vs. Ti).

To gain further knowledge of how the Ica-loaded substrates can affect macrophage activation, the secretion levels of several pro-inflammatory mediators were analysed. The release of IL-6, TNF-α, and MCP-1 was quantified by ELISA in cell culture media collected after 24 h of culture (Figure 8). The results demonstrate that, under LPS stimulation, the level of inflammatory protein markers secreted by RAW 264.7 cells increased remarkably when compared with that of the group grown in the absence of LPS. In addition, the secretion of the pro-inflammatory cytokine IL-6 could not be detected under standard culture conditions, and only after LPS stimulation did the levels increase significantly enough to be measured by the in vitro assay. Moreover, in the growth medium of the macrophages seeded directly on the surface of the TNT sample, the levels of IL-6 released were slightly lower compared to the flat Ti support and significantly down-regulated in the culture media of cells in contact with the Ica-loaded surface. Furthermore, TNF-α displayed a similar pattern of expression in culture media derived from macrophages grown onto Ti, TNT, and TNT-DP-Ica surfaces. As for MCP-1, the expression level was similar in the culture supernatants after cell incubation on Ti and TNT surfaces in both culture conditions. However, a significant reduction of MCP-1 protein expression by macrophages grown on the TNT-DP-Ica support in comparison to the flat Ti and bare TNT samples could be observed. Altogether, these data indicate that cells grown directly onto the TNT-DP-Ica substrate secrete lower levels of inflammatory mediators such as IL-6 and MCP-1.

**Figure 9:**
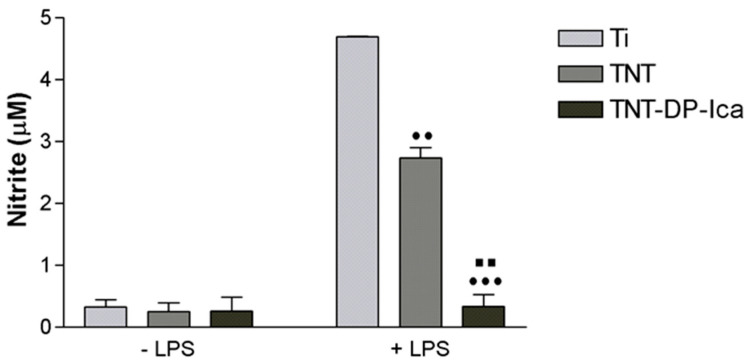
Nitrite concentrations in the cell culture media of macrophages grown on test surfaces as assessed by the Griess reaction. Under LPS-stimulation, the cells grown in contact with the flat Ti substrate secreted higher amounts of NO than on the nanostructured supports (TNT, TNT-DP-Ica). Data are presented as mean ± SD (*n* = 3, •• *p* < 0.01, ••• *p* < 0.001 vs. Ti; ▪▪ *p* < 0.01 vs. TNT).

Furthermore, the effects of the analysed samples on the LPS-induced NO production were examined at 24 h post-seeding through the measurement of the nitrite levels accumulated in the culture media. As seen in Figure 9, in standard culture conditions, the NO levels detected were very low and without any variation between the tested substrates. However, in the presence of the pro-inflammatory agent, all of the analysed samples were capable of stimulating the macrophages to release varying levels of NO, with the cells grown on the flat Ti support releasing the most considerable amounts of nitric oxide. On the other hand, the TNT-DP-Ica substrate was capable of eliciting the lowest levels of NO when compared to both flat Ti and bare TNT supports.

### 3.3. In Vivo Biological Performance Assessment

**Figure 10:**
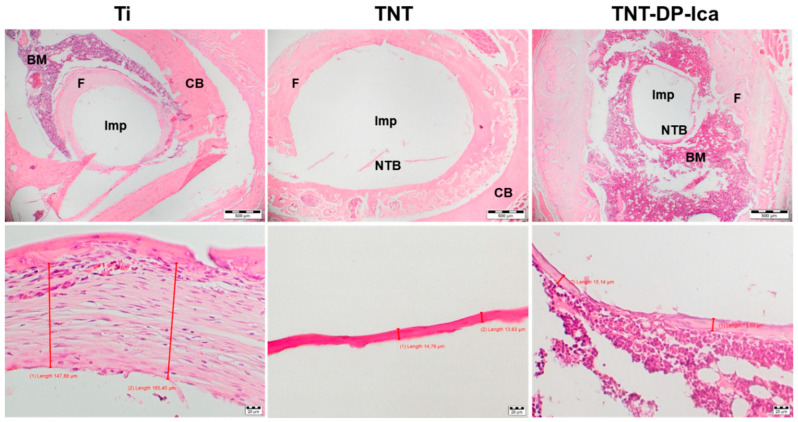
Haematoxylin-eosin (HE) staining of bone tissue surrounding the implants at 30 days post-surgery. Imp—intramedullary implant site; BM—bone marrow; CB—compact bone; NTB—newly formed trabecular bone; F—fibrosis. The white hole resulted from removing the Ti-based implants. Scale bars: 500 µm (**top images**); 20 µm (**bottom images**).

The histological analysis of the harvested bone tissue samples at 1 month post-implantation showed clear differences between the tested materials. As can be seen from Figure 10, the animal subjects with bare Ti implants showed minimal bone formation and a dense fibrous peri-implant tissue with variable thickness between 147.88–165.45 µm. In comparison to the flat Ti implant, the histological sections from animals that received TNT-modified Ti implants presented mild fibrous peri-implant tissue formation, but also bone matrix ossification with a thickness between 13.63–14.76 µm. In the case of the TNT-DP-Ica implants, minimal fibrous tissue formation and a more pronounced bone formation (thickness between 15.14–17.50 µm) could be observed. Moreover, it is worth mentioning that no inflammation could be observed for all of the analysed specimens, suggesting that the Ti-based implants are well tolerated by the organism.

**Figure 11:**
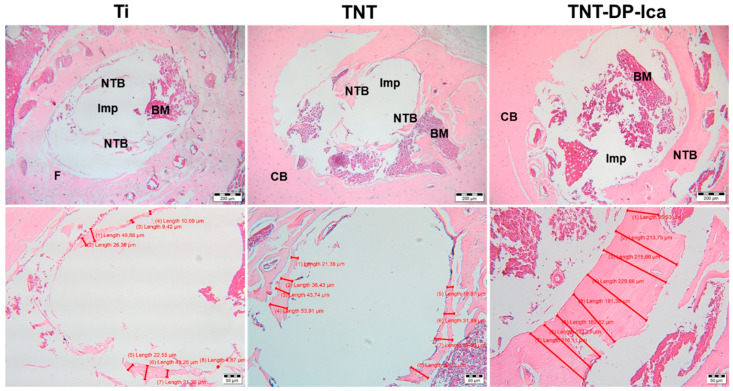
Haematoxylin-eosin (HE) staining of bone tissue surrounding the implants at 90 days post-surgery. Imp—intramedullary implant site; BM—bone marrow; CB—compact bone; NTB—newly formed trabecular bone; F—fibrosis. The white hole resulted from removing the Ti-based implants. Scale bares: 200 µm (**top** images); 50 µm (**bottom** images).

In comparison to the 1-month results, the 90-days histological sections (Figure 11) showed progressive new bone formation, visible especially in the subjects with bare Ti implants. In this case, the 30-days histological samples highlighted only the presence of a peri-implant fibrous tissue with no visible new bone formation, whereas at 90 days, the fibrous tissue was minimal, and the newly formed bone presented a thickness between 4.87–43.26 µm. Furthermore, bone formation was present in case of both surface-modified Ti-implants, with a difference of 147 µm in the averages of the newly formed bone tissue thicknesses (TNT-DP-Ica: 95.93–229.66 µm; TNT: 18.97–60.71 µm). In addition, no fibrous tissue could be observed in the subjects that received TNT-DP and TNT-DP-Ica implants. It is worth mentioning that all of the tested samples did not induce a foreign body reaction, therefore indicating that the implants continue to be well tolerated by the organism.

## 4. Discussion

Over the past half-century, the market for implantable medical devices has grown fast due to the ever-growing number of elderly patients and advances in manufacturing methods and surgical techniques. Due to their excellent biocompatibility and high resistance to corrosion, Ti and its alloys have been used worldwide for load-bearing hard tissue replacement such as orthopaedic and dental implants [51]. However, despite their outstanding properties, a major challenge for this type of biomaterial is represented by the possibility of implant failure due the bioinertness of the native TiO_2_ layer that forms on its surface, which hinders the promotion of a direct bond with the surrounding bone tissue [17,52,53]. Moreover, an ineffective osseointegration process can lead to bacterial colonization and, implicitly, infections, particularly in the case of open fractures and revision surgeries [51]. In this context, the most favourable course of action that could improve the success rate of bone surgeries is represented by various methods of surface feature modifications, regarding, e.g., the topography, roughness, charge, energy and chemical composition of Ti implants, with the purpose of ensuring favourable bone cell growth for an accelerated osseointegration [51,53,54,55,56,57]. Amongst these surface modifications, the fabrication of TNTs via electrochemical anodization stands out due to their nanoscaled surfaces which mimics the nanoarchitecture found in natural human bone tissue [53]. TNTs are self-organised nanostructures that have gained the attention of researchers due to their mechanical stability, low cost of preparation, well-defined structure, high surface area and ability to interact with various biomolecules, characteristics which turns them into excellent drug carrier platforms [53,58,59,60,61]. As mentioned before, the major challenge that needs to be overcome in this field is represented by the scarce bone formation within the tissue encompassing the material; therefore, in the last years, researchers focused on improving the functionality of TNT implants by immobilizing a wide range of biologically active molecules on the surface of the nanotubular matrices [53]. However, recent studies have revealed that by promoting only bone formation in osteoblast cells, the success rate of the implant and of the osseointegration process cannot be guaranteed. The reason behind this is that once the implant is placed into the human body, in addition to its interaction with the bone forming cells, the surface of the implant also comes into direct contact with the immune cells, mainly macrophages, therefore indicating that the osseointegration process relies heavily on the intimate cross-talk between the immune and the skeletal systems [62]. In this context, in order to expose the intimate interconnection between the skeletal and immune systems, in the current study, TiO_2_ nanotubes were grown on the Ti implant surface and loaded with Ica through an adhesive intermediate layer of DP with the purpose of exploring their effects on the in vitro inflammatory response of the murine macrophage cell line RAW 264.7, and on in vivo biological performance. The choice of Ica can be easily explained by its low cost and anti-inflammatory and anticancer capabilities [23]. In addition, Ica can promote osteoblast proliferation and the inhibition of osteoclast activity [63], with studies in the literature demonstrating not only its beneficial effects on bone forming cells but also on the immune system [64]. Moreover, recent studies have used Ica as a functional coating for various Ti surfaces, which led to an increase in the osteogenic ability of the implants [44,65]. Taking this into consideration, Ica has become an attractive candidate for the fabrication of drug delivery nanoplatforms with immunoregulatory functions and the ability to promote new bone tissue formation [63]. In addition, due to the necessity of obtaining a controlled drug release, the modified surface needed to be coated with an adhesive material that possesses excellent biological characteristics. Inspired by the amine and catechol functional groups found in the adhesive proteins of mussels, dopamine coatings have attracted the attention of researchers in the past few years [33]. Data found in the literature indicate the excellent biocompatibility of dopamine as well as its ability to promote the proliferation, differentiation, mineralization, and gene expression of osteoblast cells [33,65,66]. Furthermore, by using a single-step immersion of the TNT supports into a mild basic solution, dopamine could form a tightly adherent polydopamine layer on a wide range of surfaces through its rapid self-polymerization ability. This newly formed polydopamine coating endows the surface with a high binding strength [51,67].

Due to the direct contact between cells and the surface of various implantable devices, the physico-chemical features of Ti surfaces play an important role in directing the biological behaviour of cells. Rupp et al. [68] demonstrated that the wettability of various Ti surfaces could be greatly influenced by its roughness and morphology, while Takebe et al. [69] highlighted that the hydrophilic character of TNT structures could be slightly increased due to the easy infiltration of water into the TiO_2_ porous network, causing a decrease of contact angle value. In our study, the contact angles of the bare TNT and Ica-loaded substrates were significantly lower in comparison to that of flat Ti, in compliance with previously reported data [43]. As a means to load the biologically active molecules and drugs onto the surface of implants, the physical adsorption method has been widely applied in previous studies [40,70,71], due to its main advantage of ensuring the chemical structure and biological characteristics of the loaded molecules [43]. Our results indicate that Ica was released from the TNT-DP substrate in a sustained manner, therefore indicating that Ica was loaded successfully onto the TNT-DP surface and that its biological activity was preserved.

Macrophages hold a key role in all phases of the bone regeneration process due to the release of a wide range of factors involved in the regulation of both inflammatory and wound-healing cells [72]. Moreover, evidence is emerging that the modulation of the macrophage phenotype and its functions after contact with the biomaterial surface represents a viable strategy of obtaining desirable outcomes [73,74]. In this context, an important objective of the study was to examine the effects of Ica-loaded TiO_2_ nanotubes on macrophage behaviour in terms of cellular morphology, cell viability, and the secretion of pro-inflammatory mediators. The MTT analysis revealed that the flat Ti and bare TNT supports exhibited a higher potential to sustain cell proliferation in comparison to the Ica-loaded nanostructured surface. These results are also supported by the Live & Dead observations revealing the presence of a reduced number of macrophages in contact with the TNT-DP-Ica surface. Beside cell survival and proliferation, cellular adhesion is another important factor in the evaluation of cell behaviour towards various biomaterials. Fluorescent images of the actin-stained cells evinced differences in the cytoskeleton organization of RAW 264.7 cells grown in standard and pro-inflammatory culture conditions. Moreover, morphological features typical for the activated M1 macrophage phenotype were observed mostly on the flat Ti substrate, suggesting that this surface coupled with the LPS treatment led to a severe inflammatory response in the RAW 264.7 macrophages, whereas both the bare and Ica-loaded TNTs exhibited a protective effect against the morphological changes caused by this bacterial agent. It is worth mentioning that on the TNT-DP-Ica sample, the cells presented mostly a round morphology, typical for unstimulated macrophages. Additionally, the microscopical observations are consistent with the quantitative measurements of the spread areas and perimeters, which decreased in the following order: Ti > TNT > TNT-DP-Ica. Further, the in vitro investigation focused on the biomaterials’ ability to influence the inflammatory activity of the RAW 264.7 cells. Seconds after its introduction into the human body, synthetic biomaterial regardless of its composition and structure, will induce a series of universal immune responses which, among others, include an acute inflammatory state that is sequentially followed in a few days by a chronic inflammatory phase that can last as long as a few months, or in extreme cases a few years [75]. Depending on the type of biomaterial, the inflammatory phase can either lead to new bone formation and matrix angiogenesis or to implant failure due to fibrous tissue development as a direct consequence of the initiation of a foreign body reaction (FBR) [76]. As mentioned before, the immune cells primarily involved in the biomaterial-mediated host tissue response are represented by monocytes and macrophages [77]. Once activated, macrophages secrete and release into the microenvironment various inflammatory molecules such as cytokines, further mediating the inflammatory and wound healing responses to the presence of biomaterial [78]. Previously reported data revealed that through the influence exerted on the recruitment and differentiation of mesenchymal stem cells (MSCs) [79], and the migration and differentiation of osteoclasts [72,77], the macrophage-secreted cytokines and chemokines play an essential role in the natural bone healing process. It is generally accepted that a pro-inflammatory cytokine expression profile is correlated with a delayed healing process and bone loss [80]. Therefore, in the present work, the protein expression of the pro-inflammatory molecules TNF-α, IL-6 and MCP-1 were evaluated by ELISA and exploited as a measure of inflammatory response. TNF-α is a pro-inflammatory cytokine involved in acute inflammatory signalling induction via recruitment of the required cells for the bone regeneration process [81], while IL-6, a member of the IL-6 cytokine family, is involved in osteoclast differentiation and bone resorption [82]. However, data found in the literature suggest that the inhibition of these pro-inflammatory cytokines in the early stages of inflammation was associated with poor healing, thereby highlighting their bimodal effect in the bone regeneration process [83,84]. MCP-1 is an immunoregulatory molecule that acts as a powerful chemoattractant and activation factor for various cell types such as monocytes/macrophages, immature dendritic cells, and lymphocytes [85]. Our results show that in pro-inflammatory conditions, the level of protein expression for IL-6 was markedly enhanced by the flat Ti support in comparison to the TNT sample and the Ica-loaded surface. However, in the case of TNF-α protein expression, the levels of cytokine production displayed a similar pattern for all of the analysed samples, with no significant differences between them. As regards MCP-1 production, significantly lower levels were secreted by the RAW 264.7 macrophages grown on the TNT-DP-Ica sample compared to the flat Ti and bare TNT supports. Recent works have explored the mechanisms by which Ica targets inflammation. By suppressing p38 MAPK and activating NF-kB (Nuclear factor-kappa B) transcription factor, a decrease in the expression of TNF-α, cyclooxygenase-2 (COX-2), and iNOS was observed, thereby highlighting a potential mechanism by which Ica attenuates inflammation [78,86,87]. Moreover, it is worth mentioning that after a treatment with cyclooxygenase-2 (COX-2) inhibitors, the fracture healing process was markedly impaired [88,89,90]. Furthermore, the administration of anti-inflammatory drugs during the early stages of acute inflammation has often been associated with a delay in the bone healing process and with poor osteogenesis. In addition, in vivo studies using tibial/femoral fracture models with TNF-α receptor (p55−/−/p75−/−) knockout mice and IL-6 knockout mice, respectively, demonstrated a delay in endochondral maturation [91] and in the callus remodelling and mineralization processes [92], but these findings are scarce and need further investigation. Taking this into consideration, it could be stated that a certain but still undefined degree of acute inflammatory reaction is required for the normal bone healing process. In this context, our next objective was to investigate the relationship between the early inflammatory in vitro response of macrophages exposed to Ica-loaded Ti samples, and in vivo healing around functionalized implants. It is acknowledged that for the implant to survive after its introduction into the host body, sufficient new bone formation and a reduced inflammatory reaction are necessary. The in vivo experiments performed at 30 days post-implantation showed that both nanostructured Ti implants were more efficient in promoting the bone regeneration process when compared to the unmodified Ti implant, where a dense fibrous tissue layer was observed, which could be associated with a higher acute inflammatory response. Moreover, after 90 days of implantation, the histological analysis evinced the presence of newly formed bone tissue around all of the tested Ti-based implants with a thickness increasing in the following order: Ti < TNT < TNT-DP-Ica. However, in the case of the bare Ti implant, a layer of fibrous tissue could still be observed at the interface between the implant and the newly formed bone tissue. These results could be attributed to the nanoarchitecture and physico-chemical properties of the implant surface that led to minimal macrophage activation and implicitly to a reduced inflammatory response and improved in vivo osseointegration. Similar results were obtained in a previous in vivo study conducted on various scaffolds coated with polydopamine, which showed that the coated surfaces were capable of accelerating new bone formation and improved the osseointegration performance of the implants [93]. In addition, Hong et al. [94] reported that polydopamine can function as a biocompatible layer capable of attenuating adverse immune responses caused by the inherited characteristics of the implanted devices, whereas Bao et al. [95] demonstrated that polydopamine nanoparticles can represent a feasible strategy for reducing the specific in vivo immune response through the inhibition of ROS (reactive oxygen species) generation and of ROS-induced inflammation reactions. In addition, the phytomolecule Ica is known to promote new bone formation through the enhancement of osteoblastic differentiation and mineralization, but also through the inhibition of osteoclast activity [96]. Furthermore, in terms of inflammatory effects, Shao et al. [97] observed that Ica can significantly reduce the in vivo expression and secretion of TNF-α, IL-6 and IL-1β, thus reducing the inflammatory response caused by the presence of a synthetic biomaterial, collectively demonstrating its potential in aseptic loosening prevention and treatment.

## 5. Conclusions

In summary, the successful fabrication of the titania nanotubes and their functionalization with DP-Ica led to an improvement of surface properties in terms of surface roughness, surface energy, and corrosion resistance. The in vitro results on the effects of the Ica-loaded TNTs on macrophage behaviour indicated that the TNT-DP-Ica surface exhibited good biocompatibility with respect to cell survival, proliferation, and adhesion, but also a higher potency in suppressing acute inflammatory response. Additionally, the in vivo data revealed that at 30 days, only the modified implants were capable of promoting new bone formation, whereas at 90 days post-implantation, the presence of the regenerative process was observed in all of the three implant groups, with the TNT-DP-Ica implant showing a greater efficiency in inducing new bone formation.

## Figures and Tables

**Table 1:** Variation of the contact angle values for the three liquids analysed and surface energy.

Sample	Water		Ethylene Glycol		Dimethyl Sulfoxide		Free Surface Energy (mJ/m^2^)
	Angle	S.D.	Angle	S.D.	Angle	S.D.	
Ti	72	0.31	34	0.94	29	1.57	39.97
TNT	20	0.52	14	0.51	12	0.45	68.82
TNT-DP-Ica	35	0.69	11	0.33	8	0.36	59.27

**Table 2:** Values of resistance obtained by data fitting.

Sample	Rs (Ω)	R1 (Ω)	R2 (Ω)	χ^2^
Ti	19.4	7.27 × 10^5^	-	0.2112
TNT	18.41	4.21 × 10^5^	3.19 × 10^3^	0.0229
TNT-DP-Ica	21.84	9.84 × 10^5^	12.09 × 10^3^	0.0636

Rs-solution resistance; R_1_-resistance in parallel with CPE_1_ (constant phase element) added for the native TiO_2_ layer; R_2_-resistance for TNT and TNT-PDA-Ica supports.
